# Supplementary material for: The Interactions of the 70 kDa Fragment of Cell Adhesion Molecule L1 with Topoisomerase 1, Peroxisome Proliferator-Activated Receptor γ and NADH Dehydrogenase (Ubiquinone) Flavoprotein 2 Are Involved in Gene Expression and Neuronal L1-Dependent Functions
Source: Int J Mol Sci. 2023 Jan 20;24(3):2097. doi: 10.3390/ijms24032097 (PMC9916828; doi:10.3390/ijms24032097)
Supplement: Supplementary file 1 [file ijms-24-02097-s001.zip › ijms-2134162-supplementary.pdf]

Supplementary Material

Gabriele Loers, Ralf Kleene, Ute Bork, Melitta Schachner: The interactions of the 70 kDa fragment of cell adhesion molecule L1 with topoisomerase 1, peroxisome proliferator-activated receptor γ and NADH dehydrogenase (ubiquinone) flavoprotein 2 are involved in gene expression and neuronal L1-dependent functions

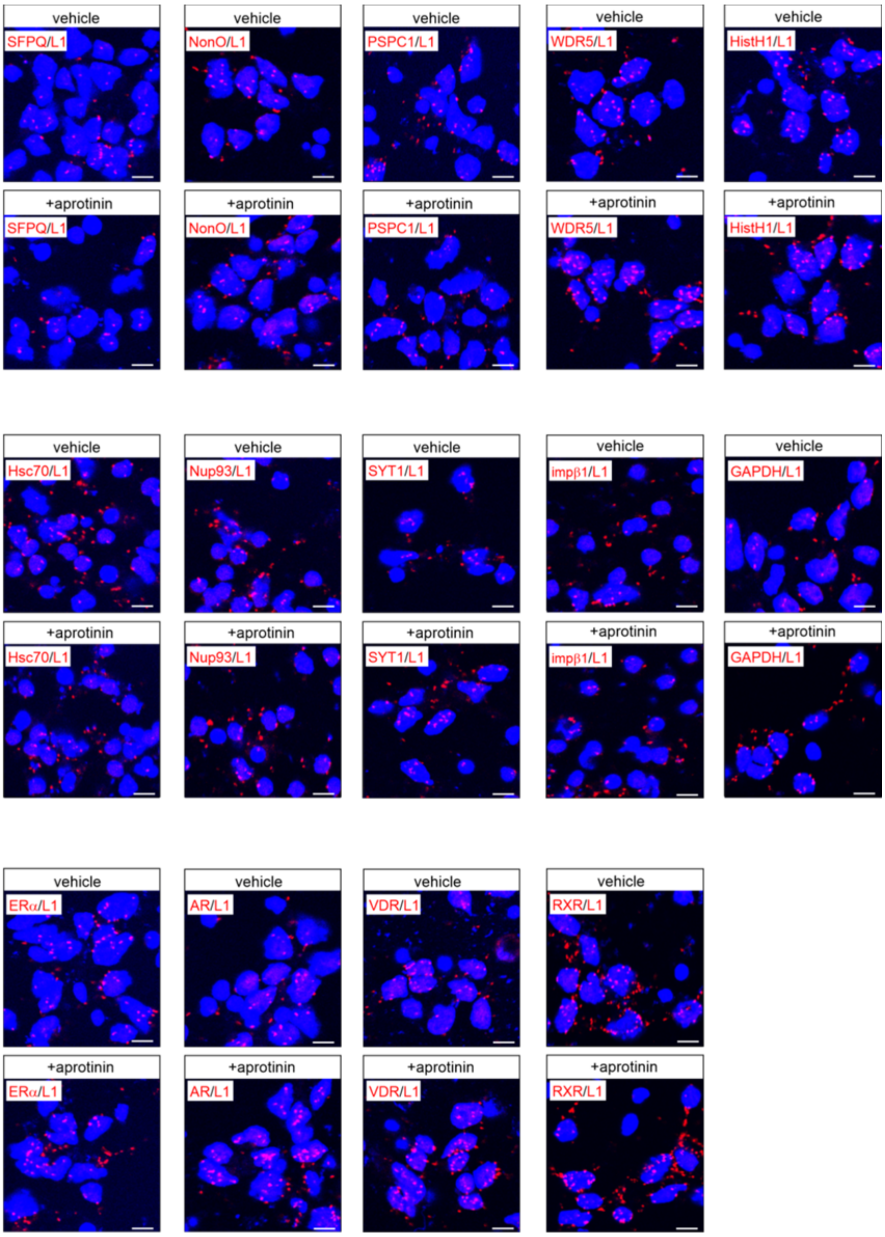

Supplementary Figure 1. L1-70 interacts with TOP1, PPARγ and NDUFV2, but not with other L1 binding partners in neurons. Cultured cortical neurons were treated with vehicle (-) or with the serine protease inhibitor aprotinin and subjected to proximity ligation with L1 antibody and antibodies against SFPQ, NonO, PSPC1, WDR5, TOP1, HistH1, Nup93, Hsc70, SYT1, impβ1, ERα, RXR, PPARγ, NDUFV2, AR, VDR or GAPDH. Representative images of vehicle- and aprotinin-treated neurons stained with the mouse L1 antibody C-2 and rabbit antibodies against SFPQ, NonO, PSPC1, WDR5, HistH1, Nup93, Hsc70, SYT1, impβ1, ERα, RXR, AR, VDR or GAPDH are shown. Nuclei are stained with DAPI. Scale bars: 10 μm.

Supplementary Table 1. Raw ELISA data corresponding to data shown in Figure 3a. TOP1 was coated and incubated with different concentrations of L1-ICD. Mean values and standard deviation of triplicates are shown for three independent experiments.

TOP1

L1 µM	1. exp mean	1. exp std	2. exp mean	2. exp std	3. exp mean	3. exp std
0.5	0.290	0.063	0.225	0.036	0.215	0.061
1	0.350	0.086	0.311	0.050	0.364	0.102
5	0.600	0.024	0.658	0.024	0.645	0.050
10	0.812	0.065	0.803	0.065	0.662	0.063
25	1.145	0.120	1.101	0.110	0.903	0.042

Supplementary Table 2. Raw ELISA data corresponding to data shown in Figure 3b. PPARγ was coated and incubated with different concentrations of L1-ICD. Mean values and standard deviation of triplicates are shown for three independent experiments.

PPARγ

L1 µM	1. exp mean	1. exp std	2. exp mean	2. exp std	3. exp mean	3. exp std
0.4	0.109	0.006	0.169	0.04	0.141	0.005
2	0.280	0.007	0.230	0.091	0.290	0.025
4	0.502	0.037	0.325	0.045	0.481	0.055
6	0.643	0.005	0.519	0.031	0.525	0.046
8	0.722	0.074	0.662	0.114	0.770	0.081
16	0.992	0.035	0.837	0.095	0.850	0.086
24	0.920	0.102	0.943	0.043	0.990	0.074

Supplementary Table 3. Competition raw ELISA data corresponding to data shown in Figure 3c. TOP1and PPARγ were coated and incubated with L1-ICD and different L1 peptides. Mean values and standard deviation of triplicates are shown for five independent experiments.

TOP1	1. exp mean	1. exp std	2. exp mean	2. exp std	3. exp mean	3. exp std	4. exp mean	4.exp std	5. exp mean	5. exp std
L1-ICD	1.492	0.190	1.548	0.173	1.349	0.130	1.484	0.120	1.200	0.110
L1-ICD+P1	0.657	0.160	0.989	0.086	0.897	0.088	0.816	0.028	0.780	0.100
L1-ICD+P2	1.821	0.047	1.488	0.098	1.287	0.101	1.315	0.071	1.188	0.068
L1-ICD+P3	1.717	0.116	1.428	0.109	1.225	0.113	1.439	0.125	1.212	0.089
L1-ICD+P4	0.128	0.073	0.727	0.089	0.504	0.092	0.600	0.058	0.384	0.059
L1-ICD+P5	1.349	0.183	1.409	0.081	1.205	0.084	1.410	0.237	1.020	0.132

PPARγ	1. exp mean	1. exp std	2. exp mean	2. exp std	3. exp mean	3. exp std	4. exp mean	4.exp std	5. exp mean	5. exp std
L1-ICD	0.769	0.052	0.979	0.120	0.856	0.053	0.636	0.106	0.848	0.043
L1-ICD+P1	0.455	0.023	0.375	0.044	0.363	0.030	0.334	0.031	0.317	0.044
L1-ICD+P2	0.828	0.061	0.941	0.094	0.830	0.108	0.715	0.057	0.893	0.082
L1-ICD+P3	0.900	0.100	0.859	0.176	0.839	0.058	0.642	0.083	0.907	0.144
L1-ICD+P4	0.390	0.038	0.366	0.080	0.359	0.065	0.265	0.067	0.417	0.096
L1-ICD+P5	0.846	0.038	0.913	0.040	0.950	0.077	0.642	0.043	0.814	0.065
